# Dengue Virus Activates Polyreactive, Natural IgG B Cells after Primary and Secondary Infection

**DOI:** 10.1371/journal.pone.0029430

**Published:** 2011-12-22

**Authors:** Thavamalar Balakrishnan, Dennis B. Bela-Ong, Ying Xiu Toh, Marie Flamand, Shamala Devi, Mickey B. Koh, Martin L. Hibberd, Eng Eong Ooi, Jenny G. Low, Yee Sin Leo, Feng Gu, Katja Fink

**Affiliations:** 1 Singapore Immunology Network, Agency for Science, Technology and Research A*STAR, Singapore, Singapore; 2 Novartis Institute for Tropical Diseases, Singapore, Singapore; 3 Department of Virology, Institut Pasteur, Paris, France; 4 Department of Medical Microbiology, Faculty of Medicine, University Malaya, Kuala Lumpur, Malaysia; 5 Blood Services Group, Health Sciences Authority, Singapore General Hospital, Singapore, Singapore; 6 Genome Institute of Singapore, Agency for Science, Technology and Research A*STAR, Singapore, Singapore; 7 Duke-NUS Graduate Medical School, Singapore, Singapore; 8 Department of Infectious Diseases, Communicable Disease Centre, Tock Seng Hospital, Singapore, Singapore; 9 Department of Haematology, St George's Hospital, London, United Kingdom; University of Rochester, United States of America

## Abstract

**Background:**

Dengue virus is transmitted by mosquitoes and has four serotypes. Cross-protection to other serotypes lasting for a few months is observed following infection with one serotype. There is evidence that low-affinity T and/or B cells from primary infections contribute to the severe syndromes often associated with secondary dengue infections. such pronounced immune-mediated enhancement suggests a dengue-specific pattern of immune cell activation. This study investigates the acute and early convalescent B cell response leading to the generation of cross-reactive and neutralizing antibodies following dengue infection.

**Methodology/Principal Findings:**

We assayed blood samples taken from dengue patients with primary or secondary infection during acute disease and convalescence and compared them to samples from patients presenting with non-dengue related fever. Dengue induced massive early plasmablast formation, which correlated with the appearance of polyclonal, cross-reactive IgG for both primary and secondary infection. Surprisingly, the contribution of IgG to the neutralizing titer 4–7 days after fever onset was more than 50% even after primary infection.

**Conclusions/Significance:**

Poly-reactive and virus serotype cross-reactive IgG are an important component of the innate response in humans during both primary and secondary dengue infection, and “innate specificities” seem to constitute part of the adaptive response in dengue. While of potential importance for protection during secondary infection, cross-reactive B cells will also compete with highly neutralizing B cells and possibly interfere with their development.

## Introduction

Symptomatic dengue infection is characterized by pyrexia, arthralgia, myalgia, headache, rash, vascular leakage and occasionally hemorrhage. With supportive medical care dengue fever (DF) normally resolves within two weeks, however in some cases patients develop dengue hemorrhagic fever (DHF) or the potentially fatal dengue shock syndrome (DSS). Fever lasts for two to seven days and coincides with the peak of viremia, although virus may still be detected in the blood for up to ten days after fever onset. Common clinical findings are an increased hematocrit and decreased platelet numbers [1]. Severe disease is more common in secondary infections, implicating that immune mechanisms are involved. Efforts to understand the immune basis of severe dengue have correlated T cell activation, in particular activation of cells from previous infection, with disease severity [2]. In addition, cross-reactive antibodies from a previous infection have been suggested to predispose to more severe secondary disease due to antibody-enhanced infection of dengue target cells [3], [4]. Overall it remains unclear to what extent pre-existing antibodies or T cells can be correlated with protection or exacerbation of disease. Antibody-dependent enhanced infections are potentially caused by non-neutralizing serotype cross-reactive antibodies.

Cross-reactive, low affinity antibodies are often generated when B cells are activated polyclonally, such as after infection with influenza [5], hepatitis C [6], HIV [7] and malaria [8]. Given this context, we hypothesized that the mechanism of B cell activation is critical in determining the outcome of dengue infection. Upon activation, B cells differentiate into plasmablasts that appear in the circulation between 6 and 8 days after infection. After secondary infection these plasmablasts produce almost exclusively IgG antibodies [9], [10], but little is known about the role of plasmablasts in primary infections due to difficulties in detecting the low numbers of antigen-specific plasmablasts and logistical constraints in obtaining early patient material.

It is well established from experiments with human volunteers that infection with one dengue serotype confers protection to all four serotypes for a limited period of a few months, after which protection becomes serotype-specific [11]. The pool of antibodies produced during acute dengue infection thus seems to be protective due to the diversity and large quantity of antibodies.

In this study, we investigated which components of the human B cell response comprised serotype cross-protection. We analyzed samples from a cohort of patients experiencing acute fever due to primary or secondary dengue infection or due to an unrelated cause. Using fresh whole blood samples we found a significant B cell activation capacity of dengue virus. A transient appearance of plasmablasts and plasma cells was observed by flow cytometry and was most pronounced during secondary infection, which could be explained by the re-activation of cross-reactive memory B cells. However even after primary infection virus-specific IgG appeared early, and IgM antibodies contributed less than IgG to virus neutralization. We hypothesize that poly-reactive B cells of the IgG isotype are specifically triggered by dengue virus and account for short-term cross-protection.

## Results

### Strong B cell activation and plasmablast formation after dengue infection

**Figure 1:**
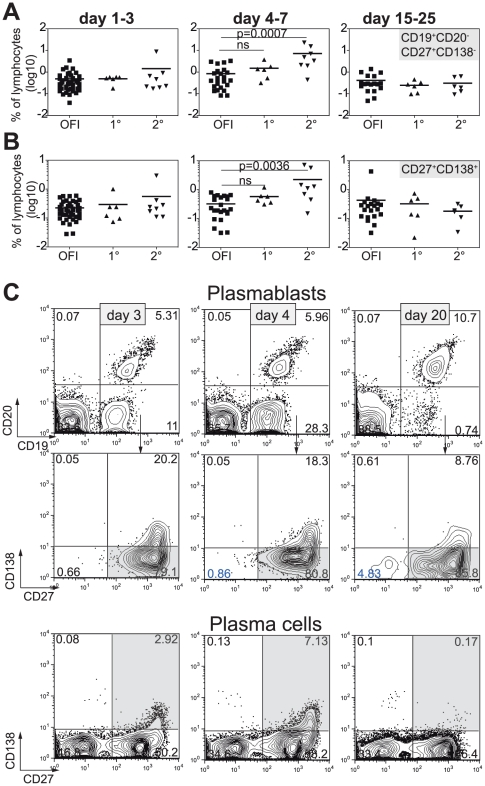
Polyclonal B cell activation after dengue infection. Fresh blood samples of dengue and control fever patients were analyzed by flow cytometry at 1–3, 4–7 and 15–25 days after onset of fever. A) CD19^+^CD20^−^CD27^+^CD138^−^ plasmablasts and CD27^+^CD138^+^ plasma cells (B) as % of lymphocytes. squares: other febrile illness (OFI), triangles: primary or secondary dengue infection. Each symbol represents one patient, lines indicate the mean. A Mann-Whitney test was used for statistical analysis. C) Gating strategy for plasmacells and plasmablasts. One secondary dengue patient with high plasma blast formation is shown. Blood was taken 3, 4 and 20 days after onset of fever.

To assess the extent and duration of B cell activation and the consequent differentiation into antibody-secreting cells during dengue infection, blood samples for analysis were taken from fever patients at the time of presentation at the clinic, at defervescence and 15–25 days after onset of fever (Table 1). Patients were retrospectively grouped as dengue- RT-PCR positive or negative, with the latter referred to as “control patients” for the rest of this study. Dengue patients were further grouped into primary or secondary cases based on the presence of dengue-specific IgG at the time point of fever (see methods). Four to seven days after onset of fever, secondary dengue patients possessed a significantly higher percentage of CD19^+^,CD20^−^,CD27^+^,CD138^−^ plasmablasts [12] (p = 0.0007) compared to control patients (Fig. 1A). Furthermore, significantly higher percentages of CD19^+^,CD20^−^,CD27^+^CD138^+^ plasma cells (p = 0.0036) were observed in dengue compared to control patients between days 4 and 7 after fever (Fig. 1B). The frequency of plasmablasts and plasma cells during primary infection appeared higher in dengue- compared to control patients. Figure 1C shows the gating strategy for plasmablasts and –cells (grey boxes), representative for one patient followed over three time points.

**Table 1:** Patient cohort.

	Total^a^	Age (years)	Sex	Fever at presentation (degC)	HCT %^c^	Plt (×10^3^/ul)^c^
**Dengue**	31	39.3±15.4	14 M, 17F	38.32±0.92	41.84±9.65	138±71
**Control group**	51^b^	37.2±16.4	27 M, 24F	38.14±0.86	47.13±10.23	198±82

a) not all experiments were done with all patient samples. The numbers of patients for each figure are indicated in the figure legend.

b) nine patients had a confirmed influenza infection and were analyzed as a separate group for Figure 1.

c) time point: 4–7days after onset of fever.

Since lymphocyte numbers are lower in dengue patients compared to control patients [13] absolute numbers of the different cell types were calculated (Table 2). Plasmablast numbers were significantly higher in dengue - compared to control patients (p = 0.033), whereas plasma cells numbers were not significantly different (p = 0.058). Interestingly, absolute numbers of plasmablasts peaked at day 4–7 in dengue patients, in contrast to day 15–25 in control patients (Table 2).

**Table 2:** Absolute numbers of plasmablasts and plasma cells.

CD19+CD20-CD27+CD138- (cells/ml)									
	day 1–3			day 4–7			day 15–25		
Diagnosis	OFI	1° dengue	2° dengue	OFI	1° dengue	2° dengue	negative	1° dengue	2° dengue
n	45	6	8	25	6	8	18	6	6
Mean	6.903	2.893	9.014	16.33	12.93	91.35	10.23	4.94	7.717
Std. Deviation	11.48	2.355	17.79	21.26	12.16	109.2	11.16	4.252	5.75
P value^a^	0.46			0.98			0.17		
P value^b^	0.41			**0.006**			0.76		

CD27+CD138+ (cells/ml)									
	day 1–3			day 4–7			day 15–25		
Diagnosis	OFI	1° dengue	2° dengue	OFI	1° dengue	2° dengue	negative	1° dengue	2° dengue
n	42	6	8	23	6	8	18	6	6
Mean	4.252	1.498	3.458	6.232	4.877	18.59	5.462	6.343	4.47
Std. Deviation	8.145	2.232	5.827	5.948	4.012	16.14	2.88	5.4	2.571
P value^a^	**0.035**			0.35			0.97		
P value^b^	0.24			0.061			0.42		

a) p value comparing OFI with 1° dengue.

b) p value comparing OFI with 2° dengue (Mann Whitney test). Longitudial samples were analyzed. Not all patients were available for the second and third time points.

Taken together, high plasmablast and plasma cell formation was observed in dengue patients and suggests production of large amounts of antibodies. Furthermore, higher numbers in secondary compared to primary dengue cases suggest re-activation of memory B cells.

### Early secretion of dengue cross-reactive IgG antibodies

**Figure 2:**
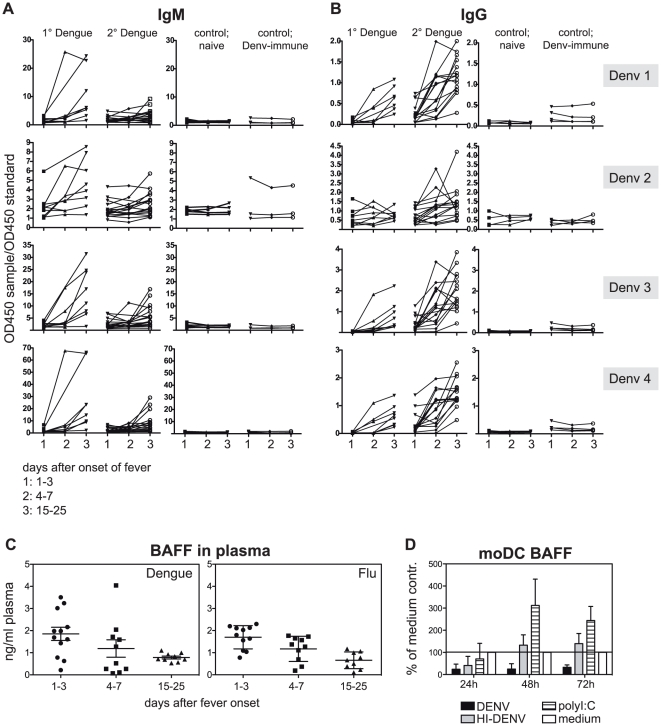
Rapid induction of cross-reactive IgG antibodies after dengue infection. Longitudinal plasma samples of dengue patients were tested by ELISA for dengue-specific IgM (A) and IgG antibodies (B). A and B) Four plasma dilutions from 1∶200 to 1∶25'000 were measured to exclude non-specific binding. For the combined illustration of all samples that were analysed the OD450 of one dilution (1∶5000 for IgG and 1∶1000 for IgM) was divided by the OD450 of a standard serum that was included on each plate. Time points: 1 = 1–3 days after fever, 2 = 4–7 days after fever, 3 = 15–25 days after fever. The same patient samples were analyzed for DENV1–4. Binding intensity correlated for all four serotypes, i.e. high binding to DENV1 would also apply to DENV2, 3 and 4. Primary versus secondary infection status was confirmed with the commercially available Panbio ELISA kit (Inverness Medical, Australia). The experiment was repeated for selected samples, confirming the original results. C) Concentrations of BAFF were measured in the plasma of dengue and flu patients at the indicated time points. D) moDCs were infected with DENV, heat-inactivated (HI) DENV, polyI:C or medium and BAFF was measured in the supernatant at different time points after infection. Data are presented as % of medium control and are the means±SEM of two independent experiments, done in triplicates. TSV01∶medium compared to polyIC∶medium is significantly different (p>0.05, Two-way ANOVA). The source of BAFF in DENV-infected patients is therefore unlikely to be DCs.

We next assessed the specificity of the activated B cell response to dengue. Plasma samples from dengue and control patients were tested for dengue serotype reactivity on ELISA plates coated with DENV1, 2, 3 or 4 (Fig. 2). Plasma was diluted 1∶200 to 1∶25'000 and each sample was measured in duplicate and compared to a standard sample that was included on each plate for inter-plate comparison. The ratio of sample/standard for only one dilution is shown for clarity in Fig. 2. DENV-specific IgM and IgG titers increased markedly between fever (1–3 days) and early convalescence (15–25 days) (Fig. 2A and B). In many secondary cases, a peak in IgG titer was observed 4–7 days after onset of fever, with cross-reactivity against all four dengue serotypes. IgG antibody levels for primary cases increased surprisingly early, between 4–7days after onset of fever (Fig. 2B).

We next thought of dengue-specific factors that could positively influence plasmablast formation. A role for B cell activating factor BAFF for B cell activation and –survival has been demonstrated [14]. We hypothesized that BAFF could be responsible for the observed plasmablast response in dengue patients since IL-10, which is highly up-regulated during acute dengue infection [15], can induce BAFF-expression [16]. We measured BAFF levels in the plasma of dengue patients and compared them to levels in patients with confirmed influenza infection amongst our control group (Fig. 2C). BAFF concentrations were higher during acute disease compared to convalescence (Fig. 2C), whereas there was no significant difference in BAFF levels between primary and secondary patients (not shown). BAFF produced during acute disease thus possibly contributes to B cell differentiation. However, this was not specific for dengue infection, and similar levels were found in patients with flu (Fig. 2C). To further test whether DENV-infection could directly induce BAFF, monocyte-derived DCs (moDCs) were infected and secreted BAFF was measured in cell culture supernatants (Fig. 2D). moDCs were chosen because DCs together with monocytes/macrophages are a major source of BAFF amongst hematopoietic cells [17], and because DCs are targets cells for DENV infection in humans [18], [19]. However, BAFF-expression in DENV-infected cells was suppressed compared to cells treated with HI-DENV or polyI:C, suggesting that DCs are unlikely to be a source of BAFF after DENV infection.

### Broad-specificity antibodies bind and neutralize dengue virus

**Figure 3:**
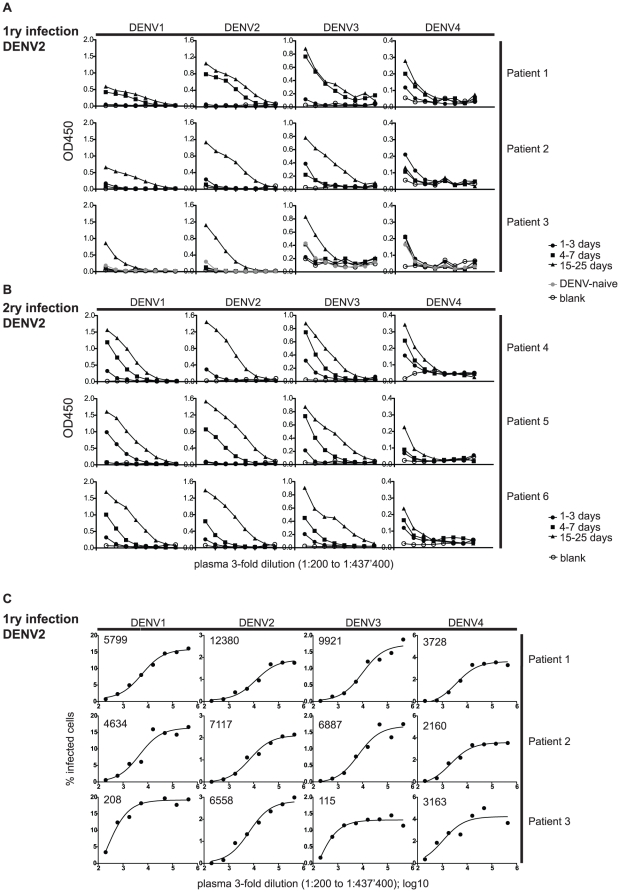
Cross-binding and cross-neutralizing IgG. Longitudinal plasma samples of three patients with primary DENV2 infection (A and C) and three patients with secondary DENV2 infection (B) were analyzed at the following time points after onset of fever: 1–3 days, 4–7 days and 15–25 days. Plasma samples were diluted three-fold over a range of 1∶200 to 1∶437'400 and analyzed by ELISA (A and B) and in a neutralization assay (C) for all four serotypes. A) ELISA with plasma from patients with primary infection with DENV2. B) ELISA with plasma from patients with primary infection with DENV2. Cross-binding to all four serotypes was observed after primary infection and was more pronounced after secondary infection. C) For the neutralization assay 1∶200 to 1∶437'400 diluted plasma samples were incubated with a constant amount of virus and the percentage of infected cells for each plasma dilution was determined by flow cytometry. EC50 values after curve fit are shown in the upper left of each graph. All curves fit with an R^2^>0.95 except for DENV4 of patient 3, where the R^2^ is 0.78. The results of both assays are representative of at least two separate experiments for all patients and time points.

It is generally accepted that neutralizing titers correlate more with protection than ELISA titers since a neutralization assay measures the capacity of antibodies to inhibit viral infection, whereas an ELISA detects all antibodies that bind to the virus. We first compared ELISA titers of the plasma of three patients with primary and three patients with secondary DENV2 infection (Fig. 3A and B). Cross-reactivity for all four dengue serotypes was observed for both patient groups but was more pronounced for secondary patients. It is well documented that neutralizing titers after secondary infection are largely serotype cross-reactive [20]. Neutralization data for patients with primary infection at early convalescence are rare though and we therefore compared the ELISA titers of the patients in Fig. 3A with neutralizing titers (Fig. 3C). A flow-cytometry based approach was used to detect infected cells and to determine the plasma dilution at which 50% virus neutralization was achieved (EC50). The neutralizing titers against DENV2 were highest (Fig. 3C), but EC50 values against DENV1 and 3 were comparable, showing that cross-reactivity is a phenomenon of both primary and secondary infection. The faster and higher cross-reactive response to all four DENV serotypes during secondary infection suggests activation or re-activation of a polyspecific pool of B cells, which rapidly produces large quantities of anti-dengue antibodies, which are superimposed on pre-existing titers generated by long-lived plasma cells.

The rapid increase of titers in primary dengue patient 1 suggested pre-existing immunity even though no pre-existing antibodies were detected by our ELISA and by the PanBio diagnostics kit.

### No hypergammaglobulinemia despite polyclonal B cell activation

**Figure 4:**
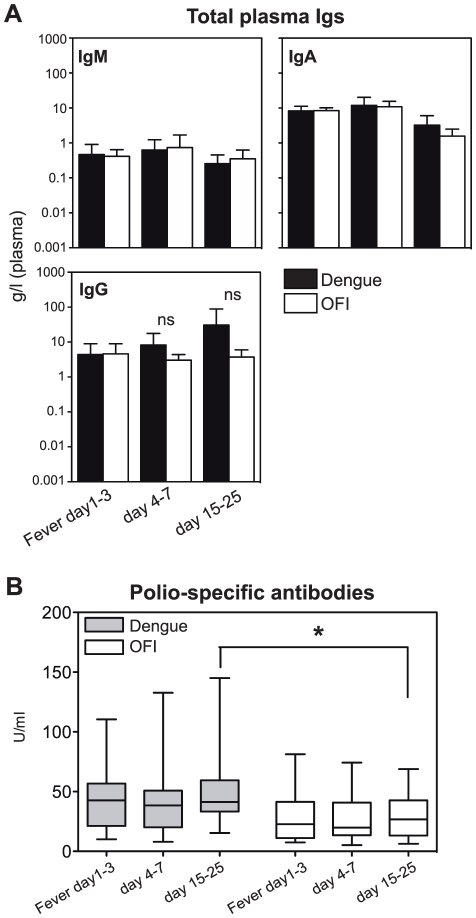
Broad specificity of dengue-induced antibodies. A) Total concentration of IgM, IgA and IgG in the plasma of dengue- and control OFI patients 1–3 days, day 4–7 and day 15–25 after onset of fever. Means±SD, n = 7–10 for dengue-negative and n = 9–16 for dengue-positive patients. A student's t test to compare dengue-positive with fever control samples was performed. B) Polio virus-specific antibodies in paired plasma samples from dengue (grey boxes) and fever control patients (white boxes) 1–3 days of fever, day 4–7 and day 15–25 after onset of fever. Data are combined from three individual experiments, n = 18 for dengue, n = 21 for OFI. A Two-Way Repeated Measures ANOVA test with Bonferroni Post-Hoc test showed a significant difference between dengue and OFI at day 15–25.

Given the cross-reactivity of both ELISA- and neutralizing antibodies, we tested whether there was an increase in total antibody concentrations, which is observed after other viral infections such as HIV known to activate B cells polyclonally [21]. There was, however, no significant difference for total IgM, IgG and IgA between dengue patients and controls (Fig. 4A). To further assess a potential polyclonal activation of B cells, polio-specific antibodies were measured during acute disease and convalescence. Dengue patients showed a slight, but significant (p<0.05) increase in polio-specific antibodies compared to control patients at 15–25 days after fever, suggesting that DENV can activate poly-specific B cells that cross-react with polio virus (Fig. 4B). The kinetics suggested production of IgG antibodies from newly activated, polio cross-reactive B cells in dengue patients, rather than re-activation of polio-specific memory B cells [22].

### Natural IgG neutralizes dengue virus

**Figure 5:**
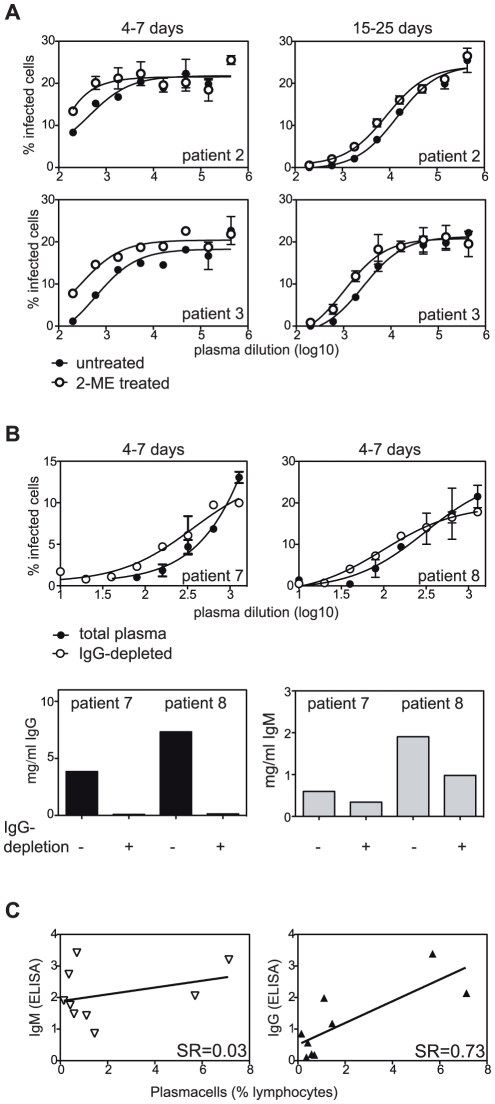
Contribution of IgM and IgG to virus neutralization. A) Plasma samples of primary patients 2 and 3 (same patients as in Fig. 3) collected during acute disease and early convalescence were treated with 0.1 M 2-Mercaptoethanol (2-ME) or medium before serial dilution and incubation with virus. 1∶200 to 1∶437'400 diluted plasma samples were incubated with a constant amount of virus and the percentage of infected cells for each plasma dilution was determined by flow cytometry. B) Plasma samples from two primary patients were IgG-depleted and analyzed as in (A). Lower panels show ELISA results to confirm IgG-depletion. 50% neutralizing titers NT50 are summarized in table 3. C) IgM and IgG binding to DENV3 (ELISA; ODsample/ODstandard of plasma samples) were correlated with % plasmacells amongst lymphocytes at day 4–7 after fever onset (refer to Fig. 1). Each symbol represents one patient, n = 9 (6 secondary and 3 primary infections). SR: Spearman R. The correlation with IgG is significant, p = 0.03.

Natural antibodies act as a first line of defense against pathogens [23], [24]. Particularly at early time points after infection we expected a substantial contribution of IgM to virus neutralization, given the increase in IgM ELISA titers at day 4–7 after fever (Fig. 2A). To determine the contribution of IgM to total neutralizing antibodies we treated plasma samples with 2-mercaptoethanol (2-ME) at a concentration that reduces disulfide bonds in IgM-pentamers while leaving antibody monomers, including IgG, intact. IgM monomers are not functional because of their low binding affinity. We found that chemical reduction of the IgM-pentamers reduced the 50% neutralizing titers (NT50) by a maximum of 50% in acute and convalescent samples (Fig. 5A and table 3). This means that already at day 4–7 after onset of fever upon primary infection IgG constitutes more than half of the neutralization. To confirm this finding we depleted IgG in plasma samples of two primary infection patients (Fig. 5B). IgG-depletion reduced the NT50 from 7175 to 364 and from 310 to 98, respectively (table 3). Even though the total IgM-concentration was also affected by depletion with Protein-G the reduction of IgM was smaller than the reduction in NT50, confirming a crucial role of IgG for early DENV neutralization and showing that the neutralizing activity cannot be attributed solely to IgM secreted from newly activated naïve B cells.

**Table 3:** 50% neutralizing titer (NT50) of 2-ME-treated (IgM-inactivated) and IgG-depleted plasma.

	4–7 days		15–25 days	
*2-ME-treated*	−	+	−	+
Patient 2	439 (R^2^ 0.82)*	200 (R^2^ 0.5)	14'393 (R^2^ 0.98)	8520 (R^2^ 0.97)
Patient 3	610 (R^2^ 0.86)	295 (R^2^ 0.9)	2603 (R^2^ 0.98)	1058 (R^2^ 0.94)

*IgG depleted*	−	+
Patient 7	7175 (R^2^ 0.98)	364 (R^2^ 0.94)
Patient 8	310 (R^2^ 0.92)	98 (R^2^ 0.99)

*NT50 (R^2^ of curve fit).

Interestingly, dengue-specific IgG ELISA titers correlated with plasma cell frequencies at day 4–7 for primary and secondary cases, whereas dengue-specific IgM ELISA titers did not (Fig. 5C). This finding indicated that early dengue-binding and -neutralizing IgG antibodies are produced by newly activated B cells that circulate as plasma cells, whereas IgM-producing cells might not appear as plasma cells in the blood.

### Serotype cross-reactivity is maintained in the memory B cell pool

**Figure 6:**
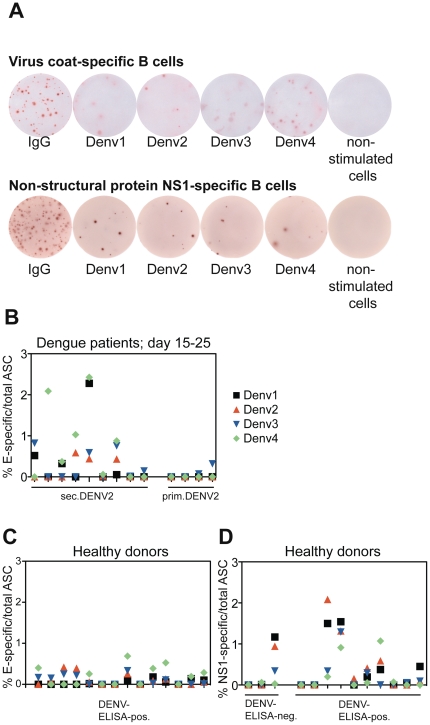
No serotype-specific selection into the memory B cell pool. A) Representative wells of the ELISPOT coated with anti-Ig, whole virus particles (E-specific B cells) or NS1 protein. Spots were detected with an anti-IgG antibody. B–D) cells were re-stimulated for six days before incubation on ELISPOT plates for the detection of memory B cells. B) Frequency of E-specific amongst total IgG-ASC in dengue patients 15–25 days after onset of fever. All patients had a DENV2 infection. Memory cells cross-reacting to two or more dengue serotypes but not necessarily binding to DENV2 were detected for most secondary patients, whereas the frequency of dengue-specific memory cells was usually below the detection limit for primary patients. C) Frequency of E-specific ASCs in healthy donors with dengue-specific IgG antibodies. D) Frequency of NS1-specific ASCs in healthy donors with or without dengue-specific IgG antibodies. B–D) Each data point in the x axes represents one donor, and values are the means of duplicates.

The early appearance of dengue serotype cross-reactive IgG antibodies in the plasma of secondary infection patients (Fig. 2B) could be either newly activated polyclonal IgG or re-activated cross-reactive memory B cells. To address this question we studied the specificity of memory B cells. We assessed the specificity for structural (E-protein) and for non-structural (NS1) protein by ELISPOT. Frozen PBMCs from secondary dengue patients and healthy donors with previous dengue infection were polyclonally stimulated according to the method developed by S. Crotty et al. [25], before overnight incubation on DENV-protein coated ELISPOT plates. Dengue-specific and total IgG antibody-secreting cells (ASCs) were analyzed (Fig. 6A). The number of IgG spots amongst unstimulated cells (if present) was deducted from the total numbers of IgG producing ASCs in all experiments shown in Fig. 6B–D. In this way we excluded plasma cells (if any) and analyzed only memory B cells.

For secondary patients we could detect E-specific memory B cell frequencies of up to 2% of total IgG memory cells during early convalescence (day 15–25 after fever), whereas specific memory could only be detected in one out of four primary patients during early convalescence (Fig. 6B). We next assessed memory B cells in healthy donors with a history of dengue infection based on anti-dengue IgG ELISA. While protection against re-infection with dengue is serotype-specific [11], we observed cross-reactivity for E-specific (Fig. 6C) and NS1-specific memory B cells (Fig. 6D). Even though the numbers of memory cells were lower in healthy donors with a history of dengue infection compared to patients at early convalescence, cross-reactivity was maintained over time. As a control for non-specific binding, seven patients without previous dengue infection were tested, and none exhibited B cell reactivity to whole DENV particles or non-structural protein 1 (NS1) protein whereas total IgG memory B cells were efficiently activated.

Interestingly, for one DENV-ELISA negative healthy donor we detected NS1-specific memory cells. Moreover, the primary patient with detectable DENV3 memory (Fig. 6B, extreme right) showed no detectable anti-dengue IgG antibodies by ELISA during the acute disease, yet rapidly increasing IgG titers at day 4–7 (Fig. 3, patient 1). These two cases show that B cell memory can still be detected in the absence of circulating antibodies.

## Discussion

Antibodies produced early during dengue infection exhibit low specificity and affinity. Cross-serotype specific protection has been demonstrated in humans for at least three months after infection by Sabin et al. during world war II. However, it cannot be concluded from those experiments whether cross-protection was mediated exclusively by antibodies and/or by innate immunity and T cells [11]. Very low incidence of dengue infections in newborns younger than 6 months in endemic areas argue for the protective value of maternal antibodies in the absence of specific T cells [26], [27].

The correlation of pre-existing immunity with disease severity is intriguing. Immune-enhancement has been observed for HIV and influenza infection [28], [29], yet an impact on the clinical outcome is known particularly for dengue disease. An infection-enhancing role for non-neutralizing antibodies has been suggested based on in vitro infection of the K562 cell line or primary monocytes in the presence of serum or monoclonal antibodies [30], [31]. However, the relevance in humans is still uncertain. Three recent studies have shed light on the B cell repertoire of human memory B cells. The common finding is that most memory B cells isolated several months after infection are dengue serotype cross-reactive [30], [32]–[34].

Few reports address the specificity of the acute B cell response during primary and secondary infection in humans [35], and the aim of this current study was to analyze the time point of B cell activation, the phenotype of B cells appearing after infection, the specificity of antibodies secreted during acute disease and, eventually, establishment of specific B cell memory.

During acute disease at day 4–7 after onset of fever we found high numbers of plasmablasts, which coincided with the appearance of dengue-specific IgG antibodies (Fig. 1 and 2). The early IgG titer implies that B cells were already isotype-switched at the time point of activation. Poly-specific B cells producing “natural antibodies” act as a first line of defense after a viral infection and have been first described in mice [24]. In mice, natural antibodies are thought to be derived mostly from B1 cells in the peritoneum and gut [36]. In higher vertebrates, natural antibodies can be of the IgM, IgG and IgA isotype [37].

The massive activation of cross-reactive B cells during acute secondary disease could result in a competition for T cell help at the expense of new neutralizing B cells, as has been shown in a mouse model of infection with lymphocytic choriomeningitis virus [38]. A concurrent B cell exhaustion could explain lower absolute plasmablast numbers in dengue patients compared to control patients at convalescence (table 2). This competition phenomenon may, at least in part, result in the increased risk associated with secondary infections, given that less efficient neutralization can be achieved against the new serotype.

Increased levels of BAFF in patients with viral infections may support plasmablast and plasmacell differentiation [14]. Hematopoietic cells are probably not the source of BAFF during dengue infection based on three observations: We were unable to detect BAFF in the supernatant of DENV-treated whole PBMCs, THP-1 monocytes produced BAFF unspecifically with or without stimuli (data not shown), and BAFF production in moDCs was even inhibited by DENV (Fig. 2D). Alternative sources of BAFF such as fibroblasts or endothelial cells [39]–[41] may therefore be more relevant in the context of DENV infection.

Next, we addressed how polyclonal activation was translated into memory since protection is serotype-specific. Dengue-specific memory B cells were successfully detected in patients with secondary infection and in dengue-immune healthy donors. Approximately 60% of the population in Singapore has antibodies against DENV indicating previous exposure to the virus. For the detection of memory B cells by ELISPOT in healthy donors we chose individuals with detectable DENV-specific IgG (Fig. 6C). However, memory B cells can exist in the absence of a detectable DENV-specific IgG titer (Fig. 6D). The frequency of memory B cells after primary infection was generally below the detection limit of our assay (1–10 in 10^6^ cells). This implies activation and expansion of broadly specific B cells during primary infection and the formation of serotype-cross-reactive memory B cells, which are re-activated and expanded during secondary infection. A recent report by Mathew et al., shows that early memory B cells 9–11 days after primary infection are serotype-specific whereas memory B cells from the same individual analyzed six months after infection are cross-reactive [35]. While serotype cross-reactivity of memory B cells after secondary infection shown in this report was in line with the mentioned study [35] we could not detect dengue-specific memory B cells 15–25days after primary infection even though numbers of total IgG-producing memory cells were as high as for secondary infection patients. The discrepancy between our study and the one by Mathew et al. might be due to the different time points analyzed and due to different B cell re-stimulation protocols. Importantly, Mathew et al did not seem to differentiate between plasmablasts and re-activated memory B cells whereas we show memory B cells only (see Material and Methods).

The binding of dengue-patient plasma to polio virus was increased compared to flu patient plasma (Fig. 4C) and supports the notion that dengue virus is a polyclonal B cell stimulator. Even though polio is given as a childhood vaccination in Singapore, we do not think that dengue infection re-activates polio-specific memory cells, because this would have resulted in an increase in polio-specific titer earlier than 15–25 days after fever [22] [42]. Binding to polio virus was not significantly higher in secondary patients compared to primary patients (not shown). Besides binding to polio antigen, we also observed increased binding of dengue patient plasma to LPS by ELISA, but not to double-stranded or single stranded DNA (data not shown). This finding suggests that potentially cross-reactive B cells are activated by dengue while B cell tolerance is maintained [43]. Despite reports of binding of NS1-specific antibodies to platelets or endothelial cells [44], we are not aware of any published incidence of autoimmune disease after dengue infection in humans.

From the data presented we suggest the following sequence of events: low-affinity, natural B cells are abundantly activated during primary dengue infection, resulting in a temporary dengue-group-specific protection for several weeks. In the long term, dengue serotype cross-reactive B cells are maintained in the memory pool, possibly alongside serotype-specific long-lived plasma cells. Memory B cells can be maintained even in the absence of detectable antibodies. Given the highly efficient re-activation of B cells as part of a phenomenon called “original antigenic sin” it will be important to monitor a balanced antibody response against all four serotypes, which is probably more relevant than absolute titers for protection.

## Materials and Methods

### Patients and healthy donors

As part of the prospective Early Dengue (EDEN) infection and outcome study in Singapore [13], adult patients (age >21 years) presenting at community primary care polyclinics with acute onset fever (>38.5°C for less than 72 h) without rhinitis or clinically obvious alternative diagnoses, were included in the study. A total of three whole blood samples were collected into EDTA-vacutainer (Becton Dickinson) tubes at recruitment (acute phase) and 4–7 days (defervescence) and 3–4 weeks after fever onset (convalescence) (Table 1). Patients were diagnosed by DENV-specific RT-PCR. DENV-specific IgM and IgG antibodies were detected by the ELISA method described here, and confirmed using the commercially available PanBio kit (Inverness Medical, Australia). DENV-RT-PCR positive patients with DENV-specific IgG antibodies at the time point of fever were classified as having secondary infections. Hematological parameters were measured using an externally quality controlled hematocytometer (Sysmex pocH 100i).

### Ethics Statement

This study was conducted according to the principles expressed in the Declaration of Helsinki. The research involving fever patients enrolled in the EDEN study was approved by the Institutional Review Board of Singapore National Healthcare Group Ethical Domain and patients gave written informed consent. Blood from anonymous healthy donors was taken with written donor consent and the use of the samples for this study was approved by the Health Sciences Authority, Singapore.

### Cell lines and virus strains

All viruses used were produced in C6/36 mosquito cells (ATCC). Following strains were used: DENV1 167 isolated from a patient in Singapore [13], DENV2 TSV01 [45], DENV3 VN32/96 and My05 34610, and DENV4 My04 31580, which are isolates from dengue patients and are a gift from Dr. Cameron Simmons, Oxford University Clinical Research Unit, Viet Nam and Prof. Shamala Devi, University of Malaya, respectively.

### ELISA

For DENV-specific ELISA, maxisorp plates (Nunc) were coated with PEG-precipitated DENV serotypes 1–4. Plates were blocked with PBS, 0.05% Tween 20 (PBST) and 3% skimmed milk. Sera and a standard containing pooled dengue-IgG positive plasma were diluted in blocking buffer 1∶200, 1∶1000, 1∶5000 and 1∶25000 and incubated on the virus-coated plates for one hour at RT before washing with PBST. Anti-human IgG-HRP (Sigma) was then added at a concentration of 1∶2000 and incubated for one hour at RT. After washing, 3,3,5,5-tetramethylbenzidine HRP substrate solution (Sigma) was added. The color reaction was stopped with 1 M HCl. To allow for inter-plate differences in coating and non-specific background binding, each sample OD is expressed relative to the OD of the standard. For clarity, the ratio of only one dilution per sample and time point is shown in Figure 2.

In some cases anti-polio antibodies were quantified as a measure of the non-dengue immune response. Polio-specific Abs were detected with an ELISA kit (AlphaScience GmbH, Riedstadt, Germany), following the company's instructions. Total amounts of IgM, IgA and IgG antibodies were detected by coating plates with anti-IgM, -IgA (Sigma) and -total Ig antibodies (Caltag), respectively. A standard was included for all isotypes (Sigma). Bound antibodies were detected using HRP-conjugated antibodies against IgM, IgA and IgG (Sigma), with TMB as the substrate.

### Neutralization assay

A flow cytometry-based neutralization assay described earlier was used with modifications [46]. BHK21 cell monolayers were grown in 96well plates. Heat-inactivated plasma samples were diluted 1∶200, 1∶2000, 1∶20'000 and 1∶200'000 in triplicates in RPMI without FCS and incubated with DENV 1 (167), 2 (TSV01), 3 (VN32/96) or 4 (My04 31580) at approx. MOI 1 for 1 h at 37degC. For some assays, equal amounts of plasma and 0.1 M 2-ME were incubated at 55°C for 1 h to reduce disulfide bonds in IgM pentamers. For depletion of IgG 150 ul of plasma were incubated with 200 ul washed Protein-G agarose beads (Millipore) overnight at 4degC on a rotating wheel. Protein-G beads were pelleted by centrifugation and the supernatant (IgG-depleted plasma) was transferred into a new tube. Treated or untreated plasma samples were dilution with medium and incubated with DENV. Plasma-virus mixtures were then transferred onto the BHK21 monolayers and incubated for 2 h at 37degC before adding RPMI, 5% FCS. After an incubation time of two or three days, cells were stained intracellularly with antibodies against NS1 and E protein and analyzed using an LSRII (Becton Dickinson). Data were analyzed using FlowJo software (TreeStar Inc.). Percentages of infected cells were plotted against the dilution factor and the EC50 was calculated with Prism5 (Graphpad Software) applying a three-parameter non-linear curve fit. Only values with a curve fit of R^2^>0.9 were considered.

### Monocyte-derived DCs

Monocytes were sorted from healthy donor PBMCs using CD14-beads (STEMCELL). Purity of CD14+ cells was 85%. Cells were incubated with human recombinant GM-CSF (50 ng/ml) and IL-4 (10 nl/ml) (both from ImmunoTools) in RPMI, 10% FCS for five days, adding fresh medium at day 3. Cells were harvested and analyzed by flow cytometry. 50–70% of cells were CD1a^+^CD14^−^ whereas the rest of the cells were CD1a^−^CD14^−^. 4×10^5^ cells per condition were stimulated in triplicates with DENV2 (TSV01) MOI2, heat-inactivated DENV2, 10 ug/ml polyI:C LMW (InvivoGen) in 0.5 ml RPMI, 10% FCS. 100 ul supernatant was collected 24 h, 48 h and 72 h later for BAFF ELISA, replenishing medium with 100 ul fresh medium without stimuli.

### B cell restimulation and ELISPOT

PBMCs were restimulated with CpG, Pokeweed Mitogen (PWM) (a kind gift from Dr. Shane Crotty, La Jolla) and Protein A from Streptococcus Aureus (Sigma) for five to six days according to the method published by Crotty et al [25]. The results were similar with fresh and frozen samples. ELISPOT plates (Millipore) were coated with PEG-precipitated virus, anti-NS1 antibody (kind gift from Dr. Marie Flamand) or anti-human Ig (Caltag) at 4°C overnight, and were blocked for two hours at 37°C with RPMI, 1% FCS. For the detection of NS1-specific B cells, NS1 produced from infected Vero cells [47] was incubated for one hour on anti-NS1 coated wells before non-specifically bound protein was washed away. Re-stimulated cells were washed twice before their addition to the blocked plates. To quantify total IgG antibody-secreting cells (ASCs), 250–25,000 cells were added to the IgG-coated wells, whereas 5×10^5^ cells were added to each dengue-coated well. Cells were incubated on the plates overnight at 37°C, 5% CO_2_. Cells were washed away with PBST the following day and anti-human IgG-HRP (Sigma) was added at a dilution of 1∶1000 and incubated for one hour at RT. Spots representing Ig-secreting B-cells were visualized with AEC substrate. Plates were washed and dried before spots were counted using an ImmunoSpot UV analyzer with BioSpot® Software (Cellular Technology Ltd). The percentage of dengue-specific memory B cells was calculated by dividing the number of dengue specific- by the total number of IgG-secreting cells. If present, spots detected in IgG-coated control wells, which were incubated with cells cultured in medium without stimuli were deducted from total IgG-secreting cells since these cells likely represent plasmablasts/cells and not memory cells.

### Flow Cytometry

Fresh whole blood or frozen PBMCs were labeled with antibodies recognizing CD20, CD27, CD19 (Biolegend) and CD138 (BD Pharmingen). For analysis, cells were resuspended in FACS buffer containing 1% formalin and analyzed using a FACS Calibur. Data were analyzed using FlowJo software (TreeStar Inc.).

### Statistical Analysis

All statistical analyses were carried out using Prism5 (Graphpad Software). The statistical test used is indicated in the figure legend. P values equal to or less than 0.05 considered significant.
